# Spatio-temporal time series forecasting with trap catch data of oriental fruit moth (*Grapholita molesta*) in peach (*Prunus persica*) orchards in South Korea

**DOI:** 10.3389/fpls.2025.1698144

**Published:** 2025-12-04

**Authors:** Steven Kim, Seong Heo

**Affiliations:** 1 Department of Mathematics and Statistics, California State University, Monterey Bay, Seaside, CA, United States; 2 Department of Horticulture, Kongju National University, Yesan, Republic of Korea

**Keywords:** *Grapholita molesta*, peach, prophet, SARIMA, smart integrated pest management, spatio-temporal time series analysis

## Abstract

The oriental fruit moth (*Grapholita molesta*, Lepidoptera: Tortricidae, OFM) is a major pest causing significant economic damage to peach (*Prunus persica*, Rosales: Rosaceae) and stone fruits in South Korea. This study aimed to describe spatio-temperal patterns of the OFM population in South Korea, forecast the OFM population using time series models, evaluate their predictive performance, and provide data-driven guidance for region-specific targeted pest management strategies. This study presents the first spatio-temporal time series analysis for predicting OFM population dynamics in peach orchards using sex pheromone trap data (Z8-dodecenyl acetate, E8-dodecenyl acetate, and Z8-dodecenol in a ratio of 88.5:5.7:1.0) collected bimonthly between May and September for ten years (2016-2025). We compared the predictive performance of Seasonal Autoregressive Integrated Moving Average (SARIMA) and Prophet models across three major peach-producing provinces in South Korea: Gyeonggi (GG), Gyeongsangbuk (GB), and Chungcheongbuk (CB). The SARIMA and Prophet models were considered because the OFM generations follow temporal trend and seasonal patterns, and these time series models are flexible to describe and predict them. The Prophet model consistently outperformed the SARIMA model in all three provinces according to multiple evaluation metrics. The time series decomposition revealed a shift from traditional multi-peak (W-shaped) patterns to single-peak patterns of the OFM occurrence, where the mass emergence usually occurs in early May. This phenological shift appears to be driven by climate changes (warmer winters and rising temperatures in the spring) coupled with varying pesticide application strategies. Spatio-temporal analysis demonstrated regional-specific variations. The province of GB maintained low OFM populations through aggressive chemical control following a major outbreak in 2016, and the province of GG showed the highest predicted occurrence in 2026. These findings highlight the importance of region-specific pest management strategies, particularly for controlling the first-generation OFM population. The predictive time series models are valuable tools for establishing smart integrated pest management systems, enabling proactive control measures tailored to regional characteristics.

## 1 Introduction

The peach (*Prunus persica*) is a perennial fruit tree belonging to the genus *Prunus* in the Rosaceae family. In South Korea, it is one of the six most popular types of fruits (apple, mandarin, grape, peach, pear, and persimmon), with an annual production value of 750.5 billion won (KREI, 2024). As of 2020, the estimated peach cultivation areas are 15,657 ha, and about 83% of the cultivation areas are in the four provinces: 7,047 ha in the province of Gyeongsangbuk (GB); 3,942 ha in the province of Chungcheongbuk (CB); 1.014 ha in the province of Jeollabuk (JB); and 911 ha in the province of Gyeonggi (GG) (KOSIS, 2025). Although peaches are not subject to import restrictions, all peaches are produced and consumed within the country because it is difficult to store for a long period of time and its quality rapidly deteriorates during the import and distribution process. As its deterioration is easily visible, peach production and quality management play a crucial role in meeting consumer demand. Notably, poor pest and disease management may result in immediate failure to meet consumer demand.

The oriental fruit moth (OFM) is one of the most damaging pests of peaches. Its known origin is Northeast Asia, and it is now found in America, Europe, and Australia (Yokoyama and Miller, 1988; Jeong et al., 2012). Both the OFM and the peach fruit moth (*Carposina sasakii*) damage fruit by burrowing into it and reduce its marketability (Lee et al., 2024). In South Korea, the OFM has been designated as a major pest since the late 1990s, and it caused significant damage to stone fruits and pome fruits. Researchers have warned that neglecting control measures can result in severe damage (Yang et al., 2001; Choi et al., 2008). The first generation of adult OFM peaks from late April to early May. The second generation peaks around mid-June, the third generation peaks around late July to early August, and the fourth generation peaks around late August to early September (Yang et al., 2001; Kim et al., 2004; Lee et al., 2024). During the production season of peaches and other stone fruits, the third and fourth generations of OFM cause damage to the fruits. To address this issue, local officials of the eight administrative regions in South Korea have conducted fruit tree pest and disease monitoring since 2016. The OFM monitoring has been conducted through field population investigations using sex pheromones or OFM bait traps.

There have been only a few studies conducting time series analyses of plant diseases and pests, and it is due to the absence of accumulated time series data on these outbreaks. In the case of COVID-19, which caused significant harm to humanity, numerous time series analysis studies were possible because of the accumulation of temporal data (Alzahrani et al., 2020; Kufel, 2020; Sahai et al., 2020; ArunKumar et al., 2021; Katoch and Sidhu, 2021; Malki et al., 2021; Roy et al., 2021; Satpathy et al., 2021; Perone, 2022). The most widely used models are the seasonal autoregressive integrated moving average (SARIMA) and the Prophet model. The SARIMA model is an extension of the ARIMA model which incorporates both seasonal and non-seasonal components (Peixeiro, 2022). It has been widely used for analyzing and forecasting data exhibiting regular, periodic seasonal variations. The Prophet is a decomposable time series model, and it is capable of analyzing complex temporal data, including non-linear trends, multiple seasonality patterns, and holiday effects (Peixeiro, 2022).

Earlier research examined population dynamics models for OFM in apple orchards, and management technologies were first introduced in Michigan during the 1970s and 1980s (Welch et al., 1978; Croft et al., 1980). These models play a crucial role in forecasting the onset of the first generation and informing pest management strategies (Croft et al., 1980; Toba and Howell, 1991). Building on field data collected from sex pheromone traps, advanced predictive models have been developed to forecast OFM emergence in practical settings (Wagner et al., 1984; Milonas et al., 2001; Knight, 2007; Stevenson et al., 2008; Ahn et al., 2012). These models utilize various mathematical functions (e.g., linear, exponential, logistic) to estimate the cumulative proportion of insects emerging during the spring generation based on degree-days accumulated above 8.14°C starting from January 1st. Related to this research, the emergence of adult OFMs in peach orchards was reported to peak four times a year (Kim et al., 2004), which is consistent with findings in apple and pear orchards (Choi et al., 2008; Yang et al., 2001, 2002). These data show temporal trend and seasonal patterns, but most of the past studies did not utilize time series models to describe the changes in the OFM population or predict the near future for practical purposes.

In this research, we aimed to (1) describe the spatio-temporal patterns of OFM in South Korea between 2016 and 2025 and identify phenological shifts in pest emergence dynamics, (2) forecast the OFM population dynamics in the major peach-producing regions of South Korea using the time series models, (3) evaluate their predictive performance, and (4) provide data-driven insights that facilitate the development of region-specific, targeted pest management strategies, which can contribute to the establishment of smart integrated pest management (IPM) systems.

## 2 Materials and methods

### 2.1 Data

The pest monitoring using sex pheromone traps has been conducted twice a month from May to September since 2016 by local government officials affiliated with the Rural Development Administration (RDA) in South Korea. The number of OFM trap catches were estimated on the first day and the sixteenth day of each month. This pest monitoring program has been continuously conducted since 2016, and the time series data are publicly available in the National Crop Pest Management System (NCPMS) operated by the RDA. For this study, the OFM monitoring data from peach orchards were downloaded from the NCPMS (https://ncpms.rda.go.kr). All surveys were conducted with the consent of orchard owners. According to the Act on Promotion of the Provision and Use of Public Data, all South Korean citizens have the right to access and utilize public data. This data source is publicly available and accessible to anyone in South Korea, and it is allowed for the research purpose.

South Korea consists of eight administrative districts: Gyeonggi (GG), Gangwon (GW), Chungcheongbuk (CB), Chungcheongnam (CN), Jeollabuk (JB), Jeollanam (JN), Gyeongsangbuk (GB), and Gyeongsangnam (GN). Agricultural technology centers, which are subordinate agencies of the RDF, designate fields in each district for the data collection. Five cities (Gyeongsan, Gimcheon, Yeongdeok, Uiseong, and Cheongdo) were selected in the province of GB, and five sites per city were surveyed annually (a total of 25 peach orchards in GB). Three cities (Yeongdong, Eumseong, and Chungju) were selected in the province of CB, and five sites per city were surveyed annually (a total of 15 peach orchards in CB). Two cities (Imsil and Jeonju) were selected in the province of JB, and five sites per city were surveyed annually (a total of 10 peach orchards in JB). Two cities (Yeoju and Icheon) were selected in the province of GG, and five sites per city were surveyed annually (a total of 10 peach orchards in GG). Similarly, one or two cities were selected in the other provinces, and five sites per city were surveyed annually.

The peach orchards within each city are randomly selected each year by local officials. That is, the pest monitoring is conducted at different orchards every year within each city to minimize potential systematic bias in the sampling. A total of 19 investigators conduct pest monitoring (one investigator per city). These investigators have a university degree in horticulture, they are regularly trained by the RDA on fruit tree pests and diseases, and they conduct pest monitoring on 95 peach orchards across the country every year following the same protocol. Pheromone traps (Delta trap, Greenagrotech, Gyeongsan, Korea) are installed in local orchards and the lure is a combination of three sex pheromone components: Z8-dodecenyl acetate, E8-dodecenyl acetate, and Z8-dodecenol, in a ratio of 88.5:5.7:1.0. The number of OFMs captured in the trap is counted, and the numbers counted across the cities are aggregated and averaged in each province. The NCPMS has published the province-level mean values of the OFM trap catches on the first day and the sixteenth day of May, June, July, and August and the first day of September in 2016 to 2025, so the data are collected nine times per year between May and September. The investigations are stopped when peach harvest is near in September, so there is no OFM monitoring on the sixteenth day of September.

### 2.2 Data preprocessing (missing values)

There were some missing the mean values of the OFM trap catches in the NCPMS. Four mean values were missing for GG (July 1, 2018; September 1, 2018; August 1, 2019; and August 16, 2019), and four mean values were missing for CB (July 1, 2018; September 1, 2018; August 1, 2019; and July 1, 2020). The missing mean values were imputed by the linear interpolation using the zoo package (Zeileis and Grothendieck, 2005) in R Version 4.5.1 (R Core Team, 2025). A missing mean value was imputed by its adjacent mean values, assuming the temporal continuity and seasonal patterns of the time series data, and the complete dataset was fitted to the SARIMA and Prophet.

### 2.3 Model fitting and forecast

The SARIMA model is denoted by SARIMA(*p*, *d*, *q*)(*P*, *D*, *Q*)*_s_* where (*p*, *d*, *q*) represent the non-seasonal parameters in the regular ARIMA model, (*P*, *D*, *Q*) represent seasonal parameters, and *s* represents the frequency which is fixed at *s* = 9. The free parameters, (*p*, *d*, *q*) for the non-seasonal components and (*P*, *D*, *Q*) for the seasonal components, were determined as follows. The augmented Dickey-Fuller (ADF) test was used to determine whether the time series data was stationary, and the autocorrelation function (ACF) was used to measure the correlation between lagged values in the time series. Given the differencing order for non-seasonal component (*d*) and seasonal component (*D*), the Akaike’s Information Criterion (AIC) was used to determine the values of non-seasonal components (*p* and *q*) and seasonal components (*P* and *Q*). This process was performed for each province. The histogram and Q-Q plot of residuals did not show severe skewness, and the correlogram did not show significant correlations after the lag zero, so the residuals appeared to be normal white noise. The Ljung-Box test on the first ten lags of residuals resulted in p-values above 0.05, so significant autocorrelations were not detected (Peixeiro, 2022). Python was used with the scikit-learn, statsmodels, numPy, and pandas packages, and R was used with the forecast package for forecasting future mean values of OFM trap catches by the SARIMA model (Hyndman et al., 2025; Hyndman and Khandakar, 2008). The default tuning parameter values were set in the auto.arima function with the seasonality option, and it searched for the best parameter values based on the AIC.

The performance of the Prophet model may be sensitive to hyperparameter values. We tested the model with different hyperparameter values, and found that it is particularly sensitive to the changepoint prior scale which controls the flexibility of the trend component. A larger value of changepoint prior scale allows more changepoints of the trend component, and a smaller value results in less changepoints (Taylor and Letham, 2018). We applied grid search from 0.001 to 50 (at 0.001, 0.01, 0.05, 0.1, 0.5, 1, 5, 10, 25, and 50) to find an optimal balance point between overfitting and underfitting. We activated yearly seasonality and deactivated weekly and daily seasonalities given the characteristics of our time series data. Python and R were used with the prophet package (Taylor and Letham, 2018, 2021).

### 2.4 Model evaluation metrics

The following four measures of prediction accuracy were considered: mean absolute error (MAE), root mean squared error (RMSE), mean absolute percentage error (MAPE), and R-squared (*R*²). For concise notations, we let *n* be the number of data points, *y_i_* be the *i*^th^ observed value, *ŷ_i_* be the *i*^th^ predicted value, and *e_i_* = *y_i_* − *ŷ_i_* be the *i*^th^ prediction error (residual) for *i* = 1, 2, …, *n*. A negative predicted value was set to zero as a negative number of OFM trap catches is practically impossible.

The MAE measures the average absolute prediction error, and it is defined as.


$$\text{MAE}=n^{-1}\sum_{i}\left|e_{i}\right|$$

A low value of MAE indicates that predicted values are close to observed values on average.

As an alternative measure, the mean square error (MSE) is the average of squared prediction errors, and it is defined as.


$$\text{MSE}=n^{-1}\sum_{i}e_{i}^{2}$$

The RMSE is defined as the square root of MSE. A lower RMSE indicates small differences between the predicted and observed values (Bi et al., 2025; Katambire et al., 2023).

The MAPE measures the average absolute prediction error relative to observed value, and it is defined as.


$$\text{MAPE}=n^{-1}\sum_{i}\left|e_{i}/y_{i}\right|\times 100\%$$

It reflects the relative error size as a percentage, with smaller values indicating smaller prediction errors (Bi et al., 2025). The MAPE was calculated after removing the observed values of zero (*y_i_* = 0).

Under the null time series model, all observed values are predicted at the overall sample mean (i.e., ${\hat{y}}_{i}=\bar{y}$). The R-squared (also referred to as the coefficient of determination) is defined as.


$$R^{2}=1-\text{MSE}/\text{MS}{\text{E}}_{0}$$

where MSE_0_ is the MSE under the null model. The value of R^2^ is between −∞ and +1, and a good predictive model has a value of R^2^ close to +1. When observed time series data have unpredictable patterns, a time series model may perform worse than the null model, and it may result in a negative value of R^2^ (Chicco et al., 2021; Kim et al., 2022).

The SARIMA and Prophet were fitted to all data (from 2016 to 2025) to describe the trend and seasonal patterns observed over the last decade; their predicted values and respective observed values were visualized; and their model fits were evaluated using the four measures. Their long-term predictions (for the next year) were tested by fitting the data from May 1, 2016 to September 1, 2024 and then predicting the data from May 1, 2025 to September 1, 2025. Forecasts for May 1, 2026 to September 1, 2026 were graphically presented. All graphs were created using the python packages: pandas, numpy, matplotlib, and seaborn. The following R packages were used to create the map of the eight districts in South Korea: ggplot2 (Wickham, 2016), dplyr (Wickham et al., 2023), and sf (Pebesma, 2018).

## 3 Results

### 3.1 GG province

**Figure 1:**
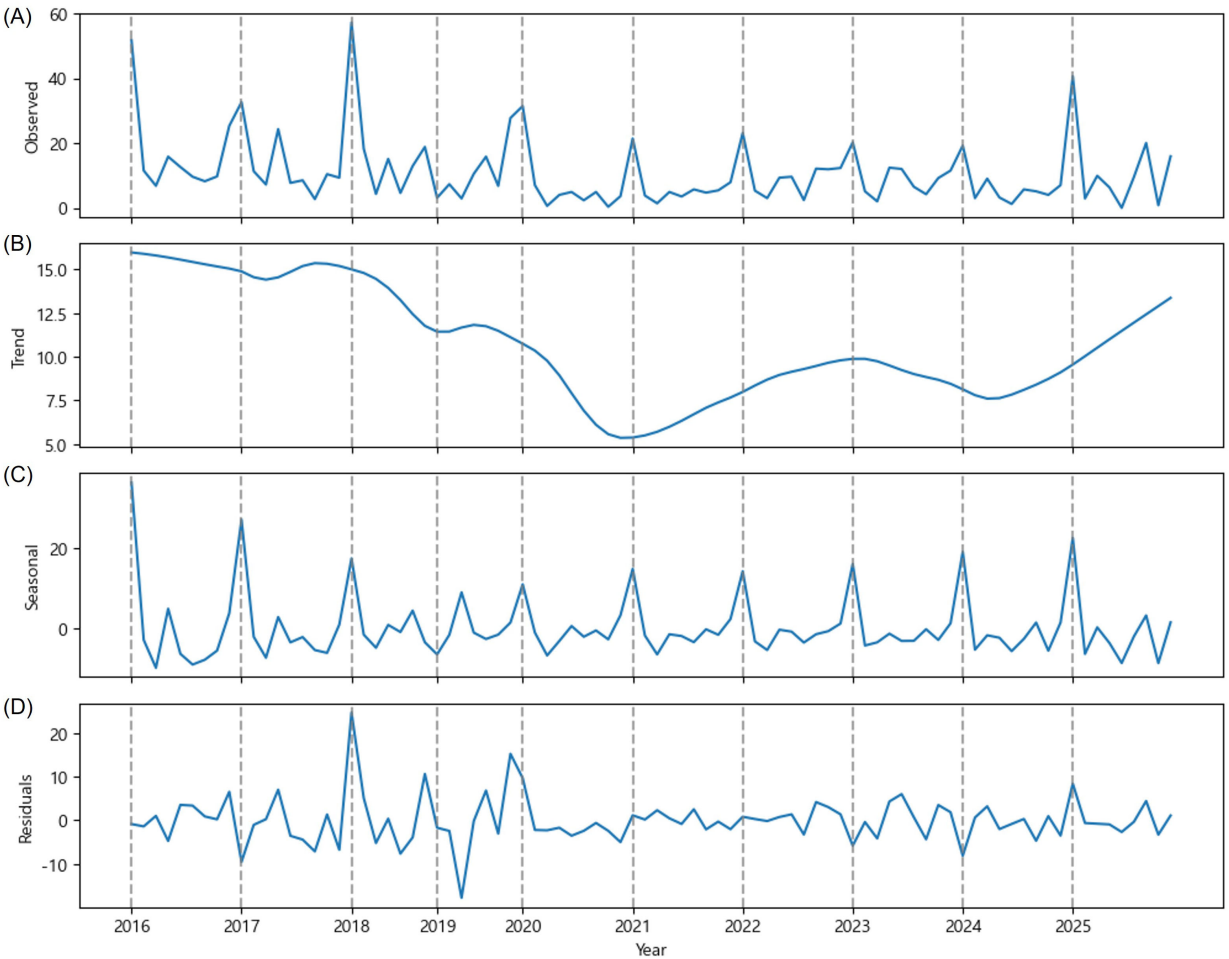
Time series decomposition of the mean value of OFM trap catches (y-axis) observed in the province of Gyeonggi (GG) from 2016 to 2025. **(A)** The observed mean value of OFM trap catches, **(B)** estimated trend component, **(C)** estimated seasonal component, and **(D)** residuals.

Based on OFM data collected from peach orchards in the province of GG, time series decomposition revealed distinct cyclical variations (**Figure 1A**). The maximum mean value of OFM trap catches was recorded on May 1st each year, and one or two minor peaks in OFM incidence occurred between June and August. Subsequently, an increase in the OFM population was observed in September. That is, the OFM data exhibited a W-shaped pattern with three to four peaks within a year from May to September. However, this cyclical pattern gradually diminished over time, and the annual outbreak has become a single peak in May.

According to the estimated trend, the mean value of OFM trap catches in the province of GG declined sharply in 2020, and started to increase in 2021 (**Figure 1B**). This declining trend likely reflects the cumulative effects of intensified pest management practices implemented following the major outbreaks in 2016 and 2018. Notably, the trend has increased since 2021, and the estimated trend reached 13 (mean value of OFM trap catches) by September 2025. This U-shaped trend pattern suggests a potential relaxation in pest pressure control or adaptation of OFM populations to management strategies over time. On May 1st, 2025, the mean value of OFM trap catches reached 40.6 which is the highest since 2019 (**Figure 1A**). Moreover, four distinct peaks were clearly identified in 2025. This means that pest control by peach growers had no impact on the OFM life cycle in 2025. Therefore, the OFM population is likely to increase in GG province by 2026.

As mentioned earlier, examining the seasonal trend reveals a distinct W-shaped pattern driven by the life cycle of OFM (**Figure 1C**). In other words, the seasonal decomposition clearly demonstrated a strong within-year cyclical pattern in the OFM occurrence. This estimated seasonality reflects the multi-voltine life cycle of OFM, with distinct generations occurring throughout the monitoring period. The consistency of the seasonal pattern across years indicates that OFM phenology remains relatively stable despite inter-annual variations in absolute population levels. The periodic spikes in the seasonal component align with the expected emergence timing of successive OFM generations, with the most prominent peaks corresponding to the first generations, which are critical periods for pest management intervention. The residuals showed no apparent systematic patterns around zero (**Figure 1D**). However, the notable residual spikes in 2018 and 2019 imply unexpectedly high numbers of OFM trap catches deviating from the estimated trend and seasonal patterns. These anomalies may be attributable to extreme weather events, localized pest management, or other environmental factors that temporarily disrupted normal OFM population.

**Figure 2:**
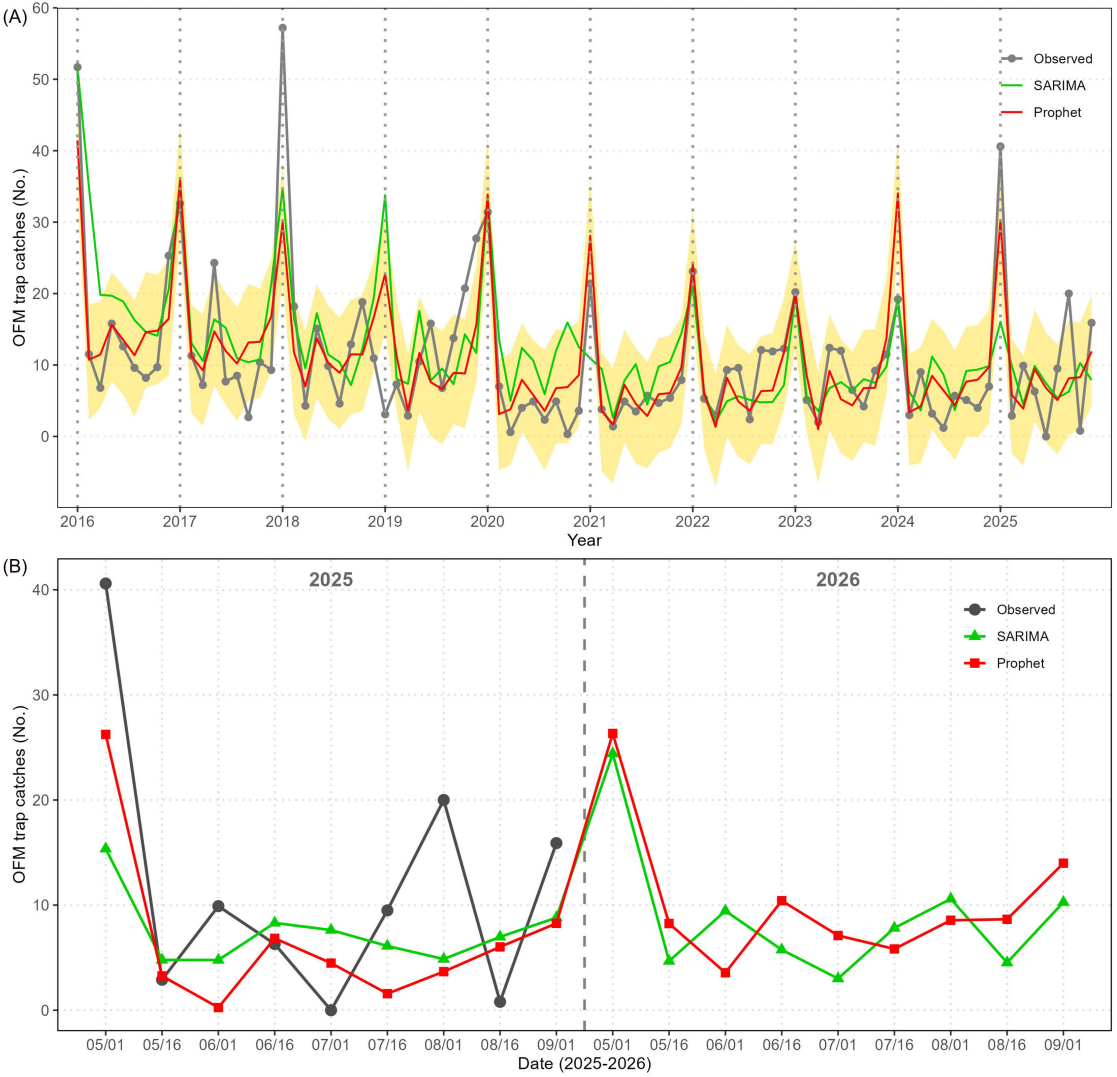
The comparison of time series models (SARIMA and Prophet) and observed mean value of OFM trap catches in the province of Gyeonggi (GG). **(A)** Full time series of the mean value of OFM trap catches from 2016 to 2025 (gray), followed by the fitted values of SARIMA (green) and Prophet (red). Shaded regions represent prediction intervals by the Prophet (shaded). The vertical dashed lines indicate new years. **(B)** Validation results of 2025 and predictions of 2026 by the two models.

The observed number of OFM trap catches and the predicted values of the Prophet and SARIMA from 2016 to 2025 are plotted in **Figure 2A**, and the shaded region represents 80% prediction intervals calculated by the Prophet model. As summarized in **Table 1**, the Prophet fitted the observed data better than the SARIMA, and its predicted values for 2025 (based on the data until 2024) were closer to the actual observed values than SARIMA’s. Neither Prophet nor SARIMA could predict the record high peak in early May 2018 and no peak in early May 2019, but since then the Prophet has accurately described the outbreak timings and magnitudes. The observed peak in early May 2025 was better predicted by the Prophet than the SARIMA (using the data until 2024), and both Prophet and SARIMA predicted that there will be another outbreak in early May 2026 (using the data until 2025) as presented in **Figure 2B**. According to the Prophet, the mean value of OFM trap catches in early May 2026 is predicted at 26.3 with a 80% prediction interval of (18.8, 34.3) which is lower than the observed outbreak in early May 2025 (mean value of 40.6 OFM trap catches). In addition, the Prophet predicted a mean value of 13.9 OFM trap catches with a 80% prediction interval of (5.8, 21.9) in early September 2026 which is close to the observed mean value in early September 2025 (15.9).

**Table 1:** Evaluation metrics for the two models (SARIMA and Prophet) based on the mean value of OFM trap catches in the three provinces: Gyeonggi (GG), Chungcheongbuk (CB), and Gyeongsangbuk (GB).

Metrics	Region	Model fit (All observed data from 2016 to 2025)		Long-term prediction (One year ahead for 2025)	
		SARIMA	Prophet	SARIMA	Prophet
RMSE	GG	8.04	6.15	10.84	9.04
	CB	7.34	6.80	7.42	5.31
	GB	7.82	6.77	4.56	5.56
MAE	GG	6.01	4.34	8.19	7.39
	CB	5.06	4.60	5.14	3.74
	GB	4.70	4.16	3.47	4.93
MAPE	GG	160.73	92.74	142.37	127.51
	CB	141.03	104.62	70.57	66.22
	GB	75.80	51.19	63.64	87.52
R^2^	GG	0.371	0.633	0.181	0.431
	CB	0.491	0.563	0.087	0.533
	GB	0.465	0.598	0.203	-0.188

### 3.2 CB province

**Figure 3:**
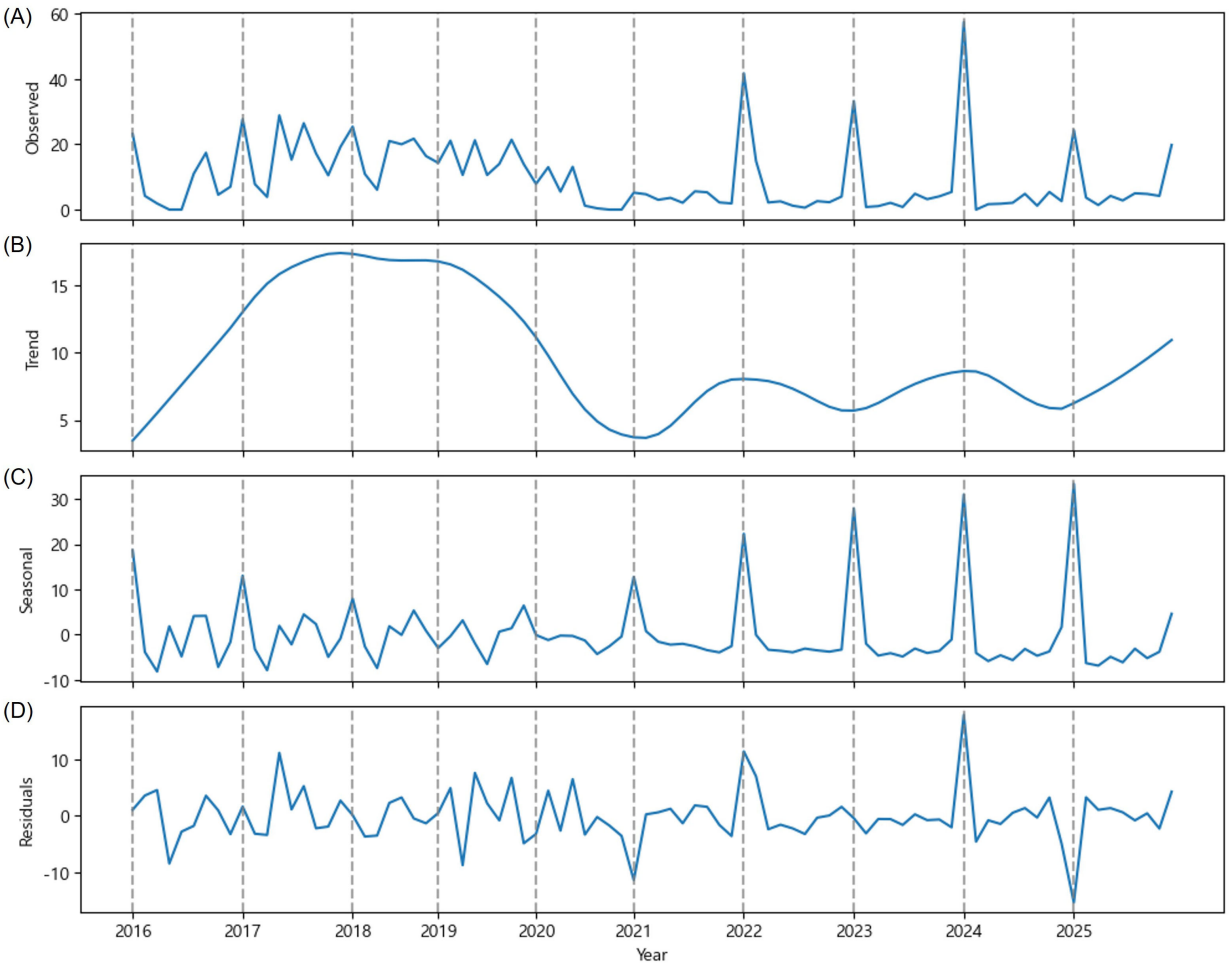
Time series decomposition of the mean value of OFM trap catches (y-axis) observed in the province of Chungcheongbuk (CB) from 2016 to 2025. **(A)** The observed mean value of OFM trap catches, **(B)** estimated trend component, **(C)** estimated seasonal component, and **(D)** residuals.

There were distinctive patterns observed over the last decade in the province of CB. There were multiple peaks a year between 2016 and 2019 which indicates persistent OFM population with the regular generational cycles. There was a transition period in 2020 and 2021 that the mean value of OFM trap catches were nearly zero, and this suppression was probably due to pest management interventions or unfavorable environmental conditions during this time. Since 2022 there has been a single sharp peak a year which is a quite distinctive pattern from the multi-peak pattern observed between 2016 and 2019 (**Figure 3A**). During the recent years exhibiting a single-peak pattern, there was a marked increase in the rapid mass emergence of first generation OFM, recording a mean value of 57.4 OFM trap catches in early May 2024, but the mean values were lower than 5 trap catches from June through September. This phenological shift represents a fundamental change in OFM population ecology in the province of CB. As shown in **Figure 3A**, pest control has failed to suppress the outbreak of the first generation in CB province. The CB region has maintained continuous OFM population control through September, but the current level shows an increasing trend (**Figure 3B**). This ascending trend suggests that OFM populations were building resistance to pest management or benefiting from favorable environmental conditions during this period. The primary cause was the failure to control the first generation OFM, which prevented the reduction of the OFM population.

According to the estimated seasonal trend, the annual multi-peak pattern has disappeared, and a single-peak pattern has become the norm (**Figure 3C**). The estimated seasonal trend reveals the first generation outbreak and suppression of the subsequent generation outbreaks. This phenological change represents a significant departure from historical OFM occurrence patterns and suggests profound changes in the factors regulating OFM populations, potentially including climate-driven shifts in voltinism or the effects of OFM generation-specific pest management strategies.

**Figure 4:**
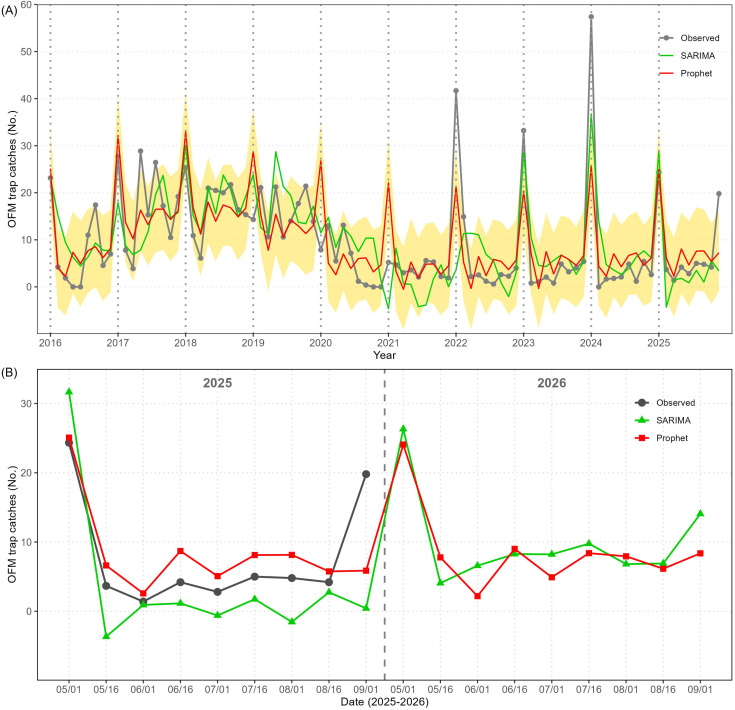
The comparison of time series models (SARIMA and Prophet) and observed mean value of OFM trap catches in the province of Chungcheongbuk (CB). **(A)** Full time series of the mean value of OFM trap catches from 2016 to 2025 (gray), followed by the fitted values of SARIMA (green) and Prophet (red). Shaded regions represent prediction intervals by the Prophet (shaded). The vertical dashed lines indicate new years. **(B)** Validation results of 2025 and predictions of 2026 by the two models.

As shown in **Figure 4A**, the Prophet model demonstrated superior adaptability to the variable population dynamics observed over time. While the SARIMA model effectively captured the general seasonal variations, it showed greater lag in responding to sudden OFM population increases. All evaluation metrics of the Prophet were better than of the SARIMA as shown in **Table 1**, and the Prophet’s predictions for 2025 (based on the data until 2024) were closer than the SARIMA’s as shown in **Figure 4B**.

When the mean value of OFM trap catches was 24.3 in early May 2025, the Prophet predicted it at 25.1 with a 80% prediction interval of (15.7, 34.1), and the SARIMA predicted it at 31.7 (21.9, 41.4) (**Figure 4B**). The magnitude of the first generation peak was predicted by the Prophet more precisely than by the SARIMA, and the observed values and Prophet’s predicted values followed the same pattern until late August 2025. Neither Prophet nor SARIMA could predict the sudden spike in early September 2025. Both Prophet and SARIMA predict similar mean values of OFM trap catches in early May 2026 and for the rest of 2026. More specifically, the Prophet predicts that the mean value of OFM trap catches will be at 24.1 in early May 2026 with a 80% prediction interval of (14.7, 32.9), and the SARIMA predicts that it will be at 26.3 (16.4, 36.2).

### 3.3 GB province

**Figure 5:**
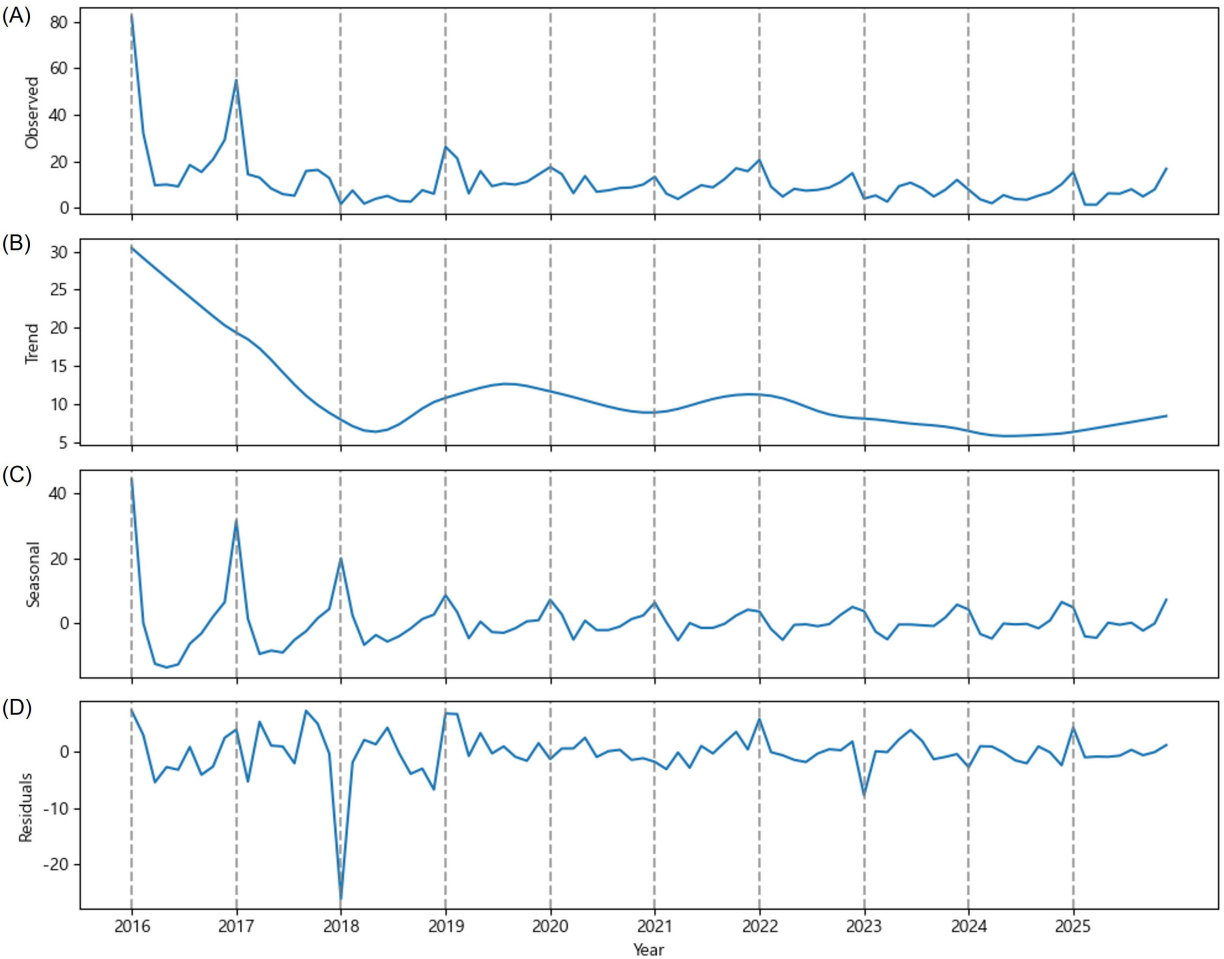
Time series decomposition of the mean value of OFM trap catches (y-axis) observed in the province of Gyeongsangbuk (GB) from 2016 to 2025. **(A)** The observed mean value of OFM trap catches, **(B)** estimated trend component, **(C)** estimated seasonal component, and **(D)** residuals.

In the province of GB, extreme outbreaks of OFM occurred in 2016 and 2017, recording the mean values of 82.1 and 54.9 OFM trap catches, respectively (**Figure 5A**). These consecutive outbreaks damaged peach orchards and triggered intensive pest management in the province. Chemical control initiated in 2017 has significantly reduced the OFM population size, and since then it has remained at a similar level to the present day. It is recognized as a successful pest management case in the nation that the OFM population has remained at the lowest level since 2018 among all provinces monitored. The estimated trend (**Figure 5B**) and seasonality (**Figure 5C**) supports the recognition. The estimated trend (the mean value of OFM trap catches) decreased from 30 in early May 2016 to nearly 5 in early May 2018 (**Figure 5B**). The magnitude of the first generation outbreak decreased from 40 in early May 2016 to nearly zero in early May 2019 (**Figure 5C**). The first generation outbreak and any subsequent outbreaks have been mild since then. Most residuals appear to be white noise, and one remarkable outlier case (below -20) was in early May 2018 when the first generation outbreak was completely suppressed by the chemical control initiated in 2017 (**Figure 5D**).

**Figure 6:**
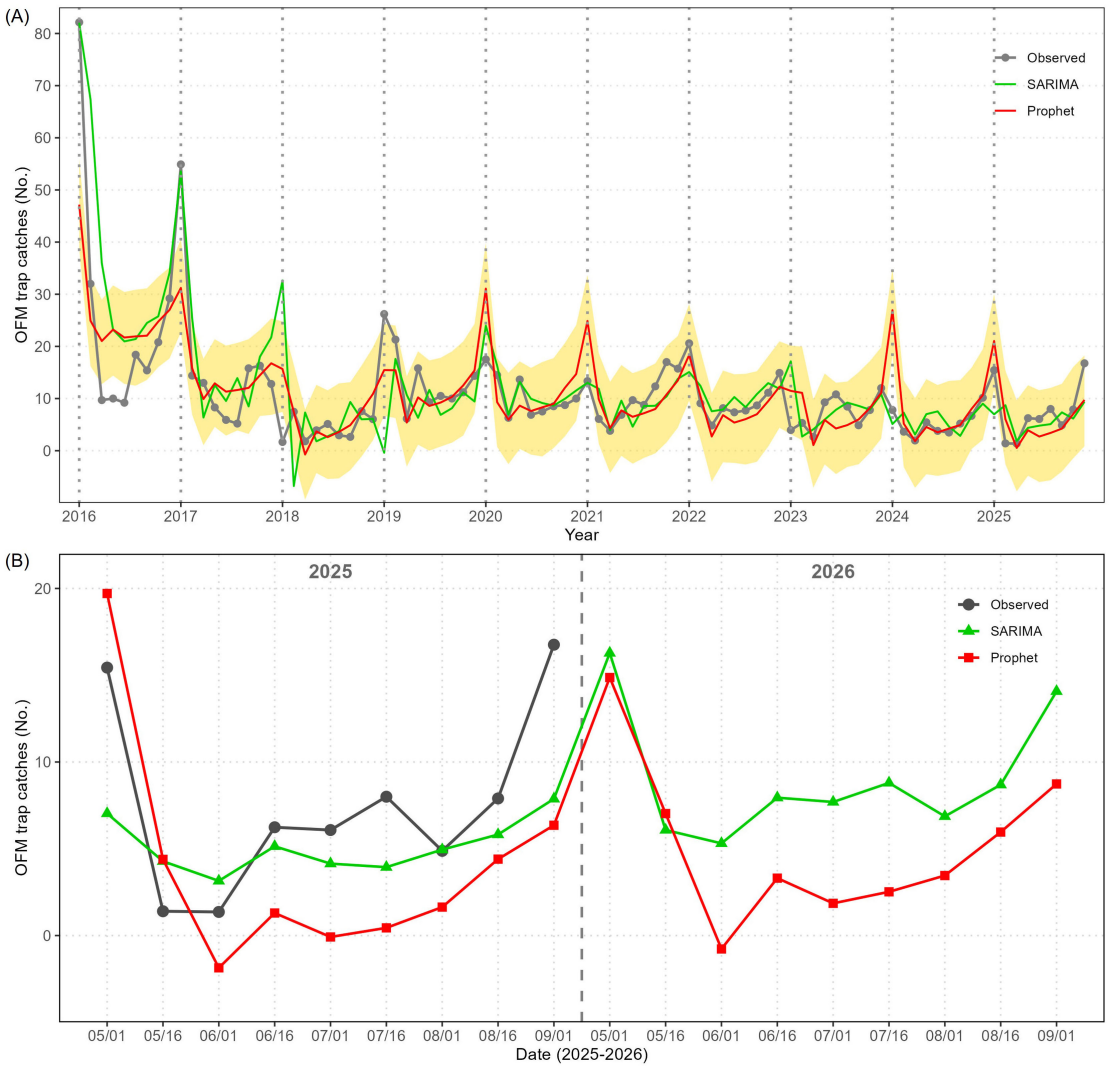
The comparison of time series models (SARIMA and Prophet) and observed mean value of OFM trap catches in the province of Gyeongsangbuk (GB). **(A)** Full time series of the mean value of OFM trap catches from 2016 to 2025 (gray), followed by the fitted values of SARIMA (green) and Prophet (red). Shaded regions represent prediction intervals by the Prophet (shaded). The vertical dashed lines indicate new years. **(B)** Validation results of 2025 and predictions of 2026 by the two models.

The SARIMA model accurately described the two extreme outbreaks in 2016 and 2017, but the Prophet model underestimated them (**Figure 6A**). It is difficult to judge visually which model fitted the observed data better, and according to the four evaluation metrics, the Prophet had a slightly better fit than the SARIMA (**Table 1**). Ironically, the OFM population controlled by the operational management in the province of GB made the Prophet’s 2025 predictions less accurate than the other provinces, and the Prophet’s predicted values for 2025 (based on the data until 2024) were not better than the SARIMA’s. The first generation outbreak was closely predicted by the Prophet, but the values between June and September were substantially underpredicted by the Prophet. It is probably because the estimated trend between 2022 and 2024 was downward (**Figure 5B**), and the Prophet predicted 2025 following the downward trend. Apparently, the SARIMA’s predictions were closer, but did not imply that the predictions were close either. The observed peak in early September 2025 was as high as in early May, and both models severely underpredicted it (**Figure 6B**).

The Prophet predicted that the mean value of OFM trap catches will be at 14.9 in early May 2026 with a 80% prediction of (6.6, 23.7), and the SARIMA predicted that it will be at 16.3 (5.9, 26.7). Both predictions are similar to the observed first generation peak in early May 2025 (the mean value of 15.4 OFM trap catches). As in the predictions for 2025, the SARIMA’s predicted values are above the Prophet’s predicted values between June and September, and the SARIMA predicted that the mean value of OFM trap catches will be at 14.1 with a 80% prediction interval of (0.9, 27.3) in early September 2026.

### 3.4 Spatio-temporal analysis

**Figure 7:**
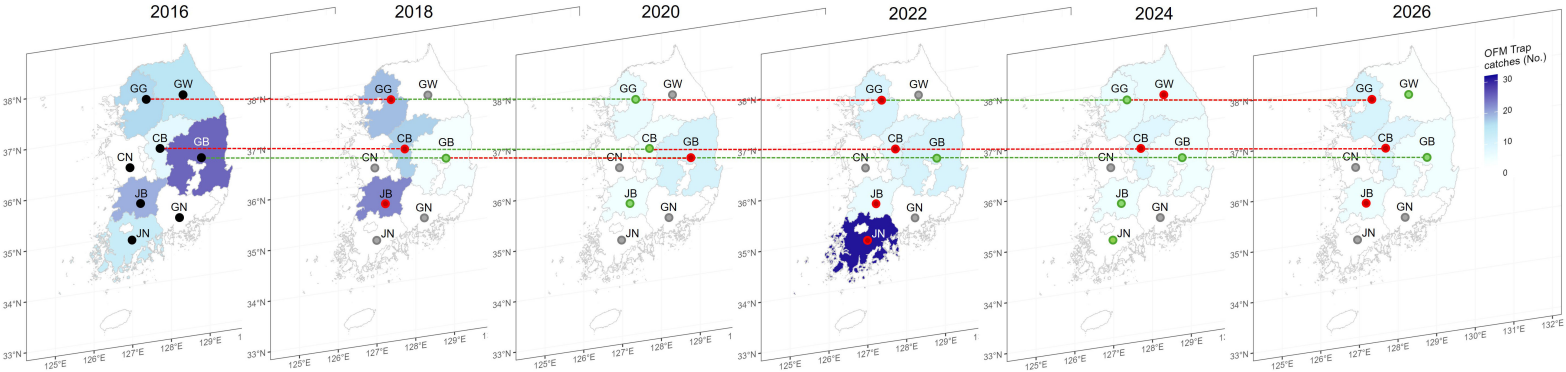
Spatio-temporal distribution of the annual average of OFM trap catches across administrative districts in South Korea. The series of maps illustrates the annual average of OFM trap catches from 2016 to 2026, with the final panel representing forecasted values in 2026 based on long-term prediction of the Prophet. Each map displays the annual average of OFM trap catches (ranging from 0 to 32 trap catches) using a blue color gradient, where darker blue indicates higher catch densities. Red dots indicate provinces where the annual average of OFM trap catches will increase in the current year compared to the previous year, while green dots indicate areas where the annual average will decrease in the current year compared to the previous year. Gray dots represent provinces with an annual average of zero trap catches. Black dots indicate the year 2016 when OFM monitoring began. Administrative regions are labeled with standard abbreviations: GG, Gyeonggi; GW, Gangwon; CB, Chungcheongbuk; CN, Chungcheongnam; JB, Jeollabuk; JN, Jeollanam; GB, Gyeongsangbuk; GN, Gyeongsangnam. The coordinate system shows latitude (33°N-38°N) and longitude (126°E-131°E) covering the Korean Peninsula study region.

To elucidate the spatial dimensions of OFM population dynamics across South Korea’s peach-producing regions, we performed the spatio-temporal analysis of trap catch data in the eight administrative provinces over the last decade. The data were visualized using the annual average of OFM trap catches (**Figure 7**). Each province is color coded according to the annual average values from 2016 to 2024 and predicted values for 2026 by the Prophet model. A darker blue region represents a higher annual average value; a red dot represents that the annual average value is higher than the previous year; a blue dot represents that the annual average value is lower than the previous year; and a gray dot represents the annual average value of zero. Since the outbreak of OFM across the entire Korean Peninsula in 2016, each province has been showing varied responses. The province of CB has shown an increasing trend since 2022, and is predicted to be increasing in 2026. In the province of GB, which produces the largest volume of stone fruits in South Korea, the OFM population is predicted to be declining in 2026 due to aggressive chemical control measures. The provinces of GG and CB provinces have shown a relatively high level of OFM population, and they are projected to be increasing in 2026. The province of GB is projected to have an annual average of 5.3 trap catches in 2026, while the province of GG is projected to have an annual average of 10.3 trap catches in 2026, which is the highest projection among the eight provinces in South Korea (**Figure 7**). Predictions for 2026 show that the provinces of CB and JB will have annual averages of 8.8 and 5.9 trap catches, respectively. Even though the provinces of GG and CB are actively controlling OFM, the outbreak in early May (single peak pattern) is becoming entrenched. To address this issue, it will be necessary to implement pest control strategies similar to that employed in GB province across GG, CB, and JB provinces.

As shown in **Figure 7**, there is not a particularly high correlation between OFM population densities in adjacent regions. For instance, the province of JN had a suddenly high average number of OFM in 2022, but its neighboring provinces (JB and GN) showed similar patterns relative to the previous years. That is, the OFM population densities do now show strong spatial autocorrelation. This weak spatial autocorrelation suggests that local factors (e.g., pest management, micro-climate, peach cultivation area) may be more important than inter-regional dispersal in determining OFM population densities. It appears that the high OFM population densities in the GG and CB regions are not due to their geographical proximity, but rather due to ineffective OFM control strategies in both areas.

Zero trap catches have been reported in most years in the province of JN, but there was a sudden spike in 2022 with an annual average of 31.1 trap catches (**Figure 7**). Such an unpredictable pattern would not be explained by time series analysis. The provinces of GN and CN have reported zero OFM occurrences for all years (2016-2025), so the time series analysis was not suitable for these provinces. The province of GW has the third smallest area of stone fruit cultivation, and there were mostly zero trap catches or missing data due to inadequate OFM monitoring, so the time series analysis was not performed for this province. Therefore, the projected average values in 2026 for the provinces of GW, CN, JN, and GN were imputed by the annual average values in 2025 or zeros in **Figure 7**.

## 4 Discussion

In this study the comprehensive spatio-temporal analysis is used to understand the OFM population dynamics in Korean peach orchards. Our results demonstrate that both time series models, SARIMA and Prophet, can describe the patterns of OFM trap catches, and the goodness of model fit varies across provinces because of provincial OFM population dynamics and pest management practices. The Prophet fitted the observed data better than the SARMA, and its predictive performance (forecasting one year ahead) was better for the provinces of GG and CB, where the population dynamics exhibited non-linear trends and complex seasonal transitions. The superior performance of the Prophet model in this study is attributed to the flexible structure of the Prophet. Particularly, the changepoint detection functionality of Prophet was advantageous for capturing abrupt changes in OFM population dynamics. Even though the Prophet fitted the data observed in the province of GB better than the SARIMA did, the Prophet substantially underestimated the OFM trap catches after the first generation peak which resulted in an inferior predictive performance for the province. Such a regional-specific predictive performance suggests that there is no one best predictive time series model for all regions.

A notable finding in this study is that the emergence pattern of the OFM has shifted from the traditional three-peak pattern (W-shaped) to a single-peak pattern over time. The shift in emergence pattern may be primarily caused by the change in the OFM life cycle due to the sustained temperature rise on the Korean Peninsula. The recent trend of shorter and milder winters, coupled with rising temperatures in springs, may cause the first generation of OFM to emerge earlier and in greater numbers. This is leading to the entrenchment of the single-peak pattern observed in early May. Previous studies have reported similar voltinism compression phenomena under warming conditions, where insect development accelerates and early generations dominate population dynamics (Tobin et al., 2008). Furthermore, the change in the OFM occurrence pattern appears to be also related to pest control history, specifically the pesticide application strategies employed by peach growers.

This phenological transition has profound implications for pest management strategy. The OFM emergence of the first generation population in early May suggests that current pest management efforts are effectively suppressing the second and third generation populations through intensive pesticide applications after May. However, persistent failures to control the first generation emergence, evidenced by higher peaks in May, indicates a critical vulnerability in the current management approach. This pattern suggests that overwintering adult populations are largely unaffected by the current pest management interventions. The targeted pest control during early spring is required to prevent OFM population buildup and subsequent generation carryover. The successful suppression in the province of GB suggests that effective OFM management requires coordinated area-wide approaches that target overwintering populations through mating disruption or precisely timed pest control (concentrated in May) instead of reactive pesticide applications during the growing season.

Spatio-temporal analysis reveals that OFM occurrence patterns vary significantly in South Korea. The weak spatial autocorrelation among adjacent regions indicates that OFM populations are largely regulated by site-specific factors rather than regional factors. While OFM adults are capable of moving across orchards, our analysis suggests that site-specific factors exert stronger influence on OFM population dynamics than regional dispersal processes. This finding underscores the significance of the intensity and timing of pest management interventions and orchard management practices in shaping OFM population dynamics. Notably, the provinces of GG and CB were predicted to have a high probability of experiencing a resurgence in OFM incidence, unlike other areas. This suggests the need for improvements to the OFM control strategies within the affected region. In the province of CB, the first generation outbreak could not be suppressed, but the populations of subsequent generations are stably managed, indicating that the effects of chemical control are partially evident. Meanwhile, the GB region has maintained low levels of OFM populations since implementing an active chemical control strategy following a major outbreak in 2016. This could be why the Prophet underpredicted the OFM trap catches in the GB region. As demonstrated in the case of GB province, systematic chemical control offers a potential pathway for effectively suppressing OFM populations. Regions experiencing early-season mass occurrence (GG and CB) may seek to adopt the pest control history implemented in GB province, optimizing pest control timing tailored to each region.

The GB province is the region where stone fruits, including peaches, are most extensively cultivated in South Korea, with the largest number of investigators conducting the OFM monitoring in the largest number of peach orchards. The provinces of CB, JB, and GG are the next largest areas of peach cultivation, and active OFM monitoring is practiced in these regions. In contrast, the OFM trap catch data from low-production regions (JN, CN, GN, GW) are unsuitable for developing time series models. This research is constrained by the data discontinuity and the occurrence of long-term zero-incidence reports within these areas, thereby precluding the application of time series analysis. There are significant limitations to determining the optimal pest control timing for each region using the time series analysis models developed through this study. The OFM occurrence data used in this study began to be collected starting in May. Not only that, but trap investigations are conducted only twice a month, making the interval too long to determine the optimal timing for pest control. Therefore, precisely forecasting the early-season mass emergence of OFM is very challenging with the current pest monitoring system. It must be based on more frequent and detailed pest monitoring. Furthermore, the temperature-driven mass emergence of OFM suggests that the model should incorporate temperature as a key factor, and we may consider a time series model in the future which can utilize covariates to explain the OFM trap catches (e.g., SARIMAX). Furthermore, the development of long-term forecasting models incorporating climate changes and pest management practice will be a key area of future research. In particular, the development of region-specific prediction models reflecting regional occurrence patterns, climate characteristics, and pest control levels is expected to play a key role in establishing a smart integrated pest management (IPM) system in the future.

## 5 Conclusions

This study is the first spatio-temporal time series analysis of the OFM population dynamics in Korean peach orchards based on OFM monitoring data over a decade (2016-2025). The time series analysis revealed the regional-specific trend and seasonal patterns of OFM trap catches. The intensive pest control after the 2016 outbreak in the province of GB is shown as a successful case. The time series trend of OFM trap catches significantly dropped shortly after the control, and relatively low peaks have been maintained until now. In the other provinces, the time series analysis showed a phenological shift from the historical W-shaped three peaks to a single peak in early May (emergence of the first generation of OFM population). Given the regional history of OFM emergences and pest management practices in South Korea, the time series results suggest (i) prioritizing early-season controls aiming the first generation of OFM, (ii) adopting a successful case of intensive pest control such as the reaction to the outbreak in the province of GB, and (iii) improving OFM monitoring systems and utilizing time series models for early-warning and decision-making stages of the smart IPM system.

## Data Availability

Publicly available datasets were analyzed in this study. This data can be found here: https://ncpms.rda.go.kr.
